# Phase I/II open-label study of the biologic effects of the interleukin-2 immunocytokine EMD 273063 (hu14.18-IL2) in patients with metastatic malignant melanoma

**DOI:** 10.1186/1479-5876-7-68

**Published:** 2009-07-29

**Authors:** Antoni Ribas, John M Kirkwood, Michael B Atkins, Theresa L Whiteside, William Gooding, Andreas Kovar, Stephen D Gillies, Oscar Kashala, Michael A Morse

**Affiliations:** 1 University of California, 11-934 Factor Building, UCLA Medical Center, 10833 Le Conte Avenue, Los Angeles, CA 90095-1782, USA; 2 University of Pittsburgh Cancer Institute, University of Pittsburgh Medical Center, Hillman Cancer Center, 5115 Centre Avenue, Pittsburgh, PA 15232, USA; 3 Division of Hematology/Oncology Beth Israel Deaconess Medical Center, MASCO 412, 375 Longwood Ave, Boston, MA 02215, USA; 4 University of Pittsburgh Cancer Institute, University of Pittsburgh Medical Center, Hillman Cancer Center, 5117 Centre Avenue, Suite 1.27, Pittsburgh, PA 15213, USA; 5 University of Pittsburgh Cancer Institute, Biostatistics Facility, Suite 325 Sterling Plaza, 201 North Craig Street, Pittsburgh, PA 15213, USA; 6 Merck KGaA, Frankfurter Str. 250, F135/129, D-64293 Darmstadt, Germany; 7 Provenance Biopharmaceuticals Corp., 830 Winter Street, Waltham, MA 02451, USA; 8 EMD Serono, Inc., One Technology Place, Rockland, MA 02370, USA; 9 Duke University Medical Center, MSRB Room 433, Box 3233, Research Drive, Durham, NC 27710, USA

## Abstract

**Background:**

To explore the biological activity of EMD 273063 (hu14.18-IL2), a humanized anti-GD2 monoclonal antibody fused to interleukin-2 (IL2), in patients with unresectable, stage IV cutaneous melanoma as measured by induction of immune activation at the tumor site and in peripheral blood.

**Methods:**

Nine patients were treated with 4 mg/m^2 ^per day of EMD 273063 given as a 4-h intravenous infusion on days 1, 2, and 3 every four weeks (one cycle). Peripheral blood was analyzed for T cell and natural killer cell phenotype and frequency, as well as levels of soluble IL2 receptor (sIL2R), IL10, IL6, tumor necrosis factor alpha and neopterin. Biopsies of tumor metastasis were performed prior to therapy and at day 10 of the first 2 cycles to study lymphocyte accumulation by immunohistochemistry.

**Results:**

Treatment was generally well tolerated and there were no study drug-related grade 4 adverse events. Grade 3 events were mainly those associated with IL2, most commonly rigors (3 patients) and pyrexia (2 patients). Best response on therapy was stable disease in 2 patients. There were no objective tumor regressions by standard response criteria. Systemic immune activation was demonstrated by increases in serum levels of sIL2R, IL10, and neopterin. There was evidence of increased tumor infiltration by T cells, but not NK cells, in most post-dosing biopsies, suggesting recruitment of immune cells to the tumor site.

**Conclusion:**

EMD 273063 demonstrated biologic activity with increased immune-related cytokines and intratumoral changes in some patients consistent with the suspected mechanism of action of this immunocytokine.

## Background

Interleukin-2 (IL2) is one of the three drugs currently approved by the U.S. Food and Drug Administration (FDA) for the treatment of metastatic melanoma. High dose IL2 induces tumor response rates of approximately 15% in patients with metastatic melanoma, with nearly half of these responses being extremely durable and leading to a seemingly cured subset of patients [1,2]. The main drawback of IL2 therapy is its toxicity, especially when administered at high doses that require hospitalization for therapy. Most patients receiving the FDA-approved high dose IL2 experience reversible grade 3 and 4 toxicities including hypotension, renal insufficiency, pulmonary edema, and cardiac arrhythmias with frequent need for continuous cardiac monitoring and administration of vasopressors such as dopamine and phenylephrine.

We hypothesized that targeted delivery of IL2 to the tumor microenvironment using immunocytokines would limit toxicity and increase efficacy of IL2-based therapies. Immunocytokines are genetically engineered fusion proteins consisting of a monoclonal antibody directed against a cancer cell surface antigen and a cytokine such as IL2 [3]. The immunocytokine EMD 273063 (hu14.18-IL2) consists of two molecules of human recombinant IL2 genetically linked to a humanized monoclonal antibody, which is directed against the diasiologanglioside GD2 (hu14.18). GD2 is a carbohydrate antigen found on the surface of human neuroectodermally-derived tumors including melanomas, neuroblastomas and some sarcomas [4]. Therefore, GD2 represents a target for the potential delivery of IL2 to the tumor site [3]. The immunocytokine is expected to maintain the activities of the monoclonal antibody that include target cell binding, effector functions such as complement-dependent cytotoxicity (CDC) and antibody-dependent cellular cytotoxicity (ADCC), while possessing cytokine function. The locally delivered IL2 may activate T and natural killer (NK) cells, which could release a secondary wave of cytokines, and activate immune effector cells.

In animal models, EMD 273063 was able to completely eradicate established lung, liver, subcutaneous, and bone marrow metastases of melanoma and neuroblastoma in immunocompetent mice bearing syngeneic tumor cells transfected to express the GD2 molecule (melanoma model), and in SCID mice reconstituted with human lymphokine-activated killer (LAK) cells and bearing human tumor xenografts (neuroblastoma) [5]. Interestingly, CD8+ T cells were required for activity of this immunocytokine in melanoma (but not in neuroblastoma), although the melanoma antigens recognized by these CD8+ T cells were not identified. Furthermore, the antitumor activity was dependent on the intact immunocytokine, since it could not be replicated by the administration of equivalent mixtures of antibody and IL2 [6].

EMD 273063 was tested in a phase I clinical trial aimed at evaluating its safety, toxicity and *in vivo *immunological effects in 33 patients with metastatic melanoma [7]. This immunocytokine was given as a 4-h intravenous infusion on days 1, 2 and 3 of week 1 at dose levels of 0.8–7.5 mg/m^2 ^per day every 4 weeks (one cycle). The best response on study was stable disease for at least 2 cycles of therapy in 8 patients. Dose-limiting toxicities defining the maximum tolerable dose (MTD) of 7.5 mg/m^2 ^per day included hypoxia, hypotension, and elevations in liver function tests. Immune activation was induced, as measured by rebound lymphocytosis, increased peripheral-blood NK cell number and activity, and increased serum levels of the soluble alpha chain of the IL2 receptor complex (sIL2R), which was observed at doses both higher (4.8 mg/m^2 ^per day) and lower (3.2 mg/m^2 ^per day) than the dose selected for evaluation in the current study. These results were replicated in a separate phase I clinical trial in a pediatric population of patients with neuroblastoma (27 subjects) and melanoma (one subject) treated with EMD 273063 [8]. Evidence of immune activation was based on increases in serum levels of sIL2R and rebound lymphocytosis. There were no major objective tumor responses, but some patients with chemotherapy-refractory neuroblastoma had periods of durable disease stabilization. In this population, the MTD of EMD 273063 was determined to be 12 mg/m^2 ^per day.

We hypothesized that the augmented immune activation detectable in peripheral blood after administration of EMD 273063 would be associated with enhanced immune cell infiltrates in melanoma lesions. Therefore, we performed this study to estimate the biologic effects of EMD 273063 at 4 mg/m^2 ^per day for 3 days as measured by the induction of immune activation in peripheral blood and at the tumor site in a pilot group of patients. The dose of 4 mg/m^2 ^was chosen for further clinical evaluation because the toxicity increased with higher doses in the prior phase I/II clinical trials, whereas there was evidence of reproducible immune activation at this dose level [7,8].

## Methods

### Study design and endpoints

Study EMR 62207-005 was a phase I/II, open-label, multi-center (4 centers in the USA) clinical trial. Prior to study initiation, the protocol and informed consent documents were approved by the Institutional Review Boards at each study center, and the study was conducted in accordance with both the provisions of the Declaration of Helsinki and Good Clinical Practice. Site monitoring included review of the accuracy of the data in the case report forms. The study planned to enroll 12 eligible patients to explore the effect of EMD 273063 on the study endpoints. This number was based on previous experience with immune analyses indicating that relevant immune responses could be detected with 9–12 patients. This clinical trial was not powered to make inferential statistical analyses. The primary study objective was to estimate the biological activity of EMD 273063 as measured by induction of immune activation in peripheral blood and at the tumor site. Secondary objectives were clinical anti-tumor activity, safety, toxicity and pharmacokinetics (PK) of EMD 273063. Toxicity grades were classified according to the NCI Common Toxicity Criteria Version 2. Objective tumor responses were assessed by the investigators using Response Evaluation Criteria in Solid Tumors (RECIST) [9].

### Patient selection

Eligible patients had histopathologically confirmed stage IV cutaneous melanoma that was not amenable to surgical treatment with curative intent, had progressed after prior therapy including IL2 and/or interferon (IFN), had a Karnofsky performance status of ≥ 70%, and had adequate organ function. Patients were enrolled at least 4 weeks after their last dose of prior therapy. Patients were to have at least 4 melanoma lesions (other than a target lesion) available for outpatient biopsies. The inclusion criteria initially required that the patients be HLA-A2-positive to allow for the assessment of CD8 responses to HLA-A2-restricted melanoma peptides. This criterion was later modified to enhance enrolment. GD2 expression by tumor cells was not an eligibility criterion because assays for GD2 surface expression were not felt to be robust at the time [10].

### Study drug administration

EMD 273063 was provided as a frozen solution in 4-mL glass vials at a concentration of 1 mg/mL, and was manufactured for EMD Serono Research Center, Inc. (Billerica, MA) and EMD Serono Biotech Center, Inc. (Billerica, MA) by Draxis Pharma Inc., Canada. EMD 273063 was diluted with 0.9% sodium chloride for injection and 0.25% human serum albumin before infusion, and administered as an intravenous infusion over 4 h at 4 mg/m^2 ^per day for 3 consecutive days every 28 days. Infusions were performed in an inpatient setting in a General Clinical Research Center. Patients were eligible for up to 4 cycles of treatment.

### Pharmacokinetics

Blood samples for PK analyses were drawn during cycles 1 and 2 as pre-dose samples taken immediately before the start of infusion, and post-dose samples collected at 2, 4, 5, 6, 8, 12, and 24 h after start of infusion on day 1. The sample taken at 4-h post-infusion corresponded to the end of infusion (EOI) sample. During cycle 2, the 12-h sample was not required. Additional pre-dose and EOI samples were taken on days 2 and 3 of both cycles. Samples were processed and analyzed for the determination of EMD 273063 in serum using a validated enzyme-linked immunosorbent assay (ELISA). Descriptive PK parameters were derived by non-compartmental and compartmental analysis using the software program Kinetica™ (Thermo Electron, Philadelphia, PA).

### Immune monitoring in peripheral blood samples

All assays on peripheral blood were performed at the Immunologic Monitoring and Cellular Products Laboratory of the University of Pittsburgh Cancer Institute Research Pavilion at the Hillman Cancer Center, Pittsburgh, PA. Patients underwent collection of peripheral blood (20–90 mL depending on the study day) pre-study, on days 1 and 10 of each cycle of therapy and at the completion of therapy. Peripheral blood mononuclear cells (PBMC) were separated by density gradient centrifugation over Ficoll gradients and cryopreserved for later analyses. The following analyses were performed as a readout of immune activation: T cell phenotyping for CD3, CD4, CD8, CD16, CD25, CD27 and CD56 by flow cytometry; intracellular granzyme B by flow cytometry as a surrogate marker of the cytotoxic potential of circulating lymphocytes; NK cytotoxic activity against the erythroleukemia cell line K562 (NK-sensitive target) as assessed by standard ^51^Chromium release assays; ADCC was determined by incubating NK cells with an NK-resistant melanoma cell line (FEMX) and EMD 273063; sIL2R, neopterin and the cytokines IL6, IL10, tumor necrosis factor alpha (TNF-α), and S100 were all measured in serum by commercially available ELISA kits (R&D Systems, Minneapolis, MN). The ELISA analyses of sIL2R, neopterin, IL6, IL10 and TNF-α were conducted with peripheral blood samples obtained on each of the first 3 days of the first 2 treatment cycles. The peripheral blood sample for baseline measurements was obtained by combining two pre-treatment samples (a screening sample and a sample obtained just before the first dose).

### Analysis of tumor biopsies

All biopsy tissue assays were performed at Genzyme Analytical Services, Los Angeles, CA. Tumor tissue specimens were obtained at initial screening and at approximately day 10 of the first 2 cycles. Sections of biopsies were snap-frozen using liquid nitrogen, embedded in epoxy, cut and stained with hematoxylin and eosin. Additional sections were embedded in paraffin and labeled with appropriate antibodies for immunophenotyping by immunohistochemistry (IHC). Assays included the density of inflammatory and immune cells; the expression of the T and NK cell cytotoxic granule granzyme B; GD2 immunostaining to define changes in the target of EMD 273063; and major histocompatibility complex (MHC) class I antigen expression. Photographs were taken with an Olympus DP10 digital camera attachment with a C-mount adapter mounted on an Olympus BX40 compound microscope with 4×, 10×, 20× and 40× power objectives. Samples were scored as positive if there were ≥ 50% of cells with 1+ or greater staining intensity (GD2, S100, or HLA-A), or ≥ 1.0 cells per high power field (cell/HPF). In addition, the relative intensity of staining (0, 1+, 2+, and 3+) and the percentage of cells with each degree of staining were also recorded.

### Statistical analysis

Exploratory analyses using descriptive statistics were performed to study the biologic activity of the study drug. For parameters in peripheral blood with 3 or more observations, the Mack-Skillings test was conducted as an omnibus test of changes over time. Mack-Skillings p values were adjusted by the step-down Bonferroni method. If an endpoint produced an adjusted p value that was less than 0.05, contrasts between specific study days were tested with the signed rank test. These included comparing days 1–10 of cycle 1 except for serum cytokines for which day 1 to day 3 comparisons were conducted for the first 2 cycles. Some immune parameters that lacked enough samples for the omnibus test were analyzed by comparing pre-treatment to cycle 1 day 10 with the signed rank test. Signed rank p values were not adjusted for multiple hypothesis tests. Semi-quantitative changes in immunohistochemical staining of tumor tissue before and after treatment were analyzed for significance with the McNemar's test.

## Results

### Patient characteristics

Between June and November 2002, 10 of the 12 originally planned patients were enrolled at 4 study sites. Enrollment was stopped early when the study drug was nearing its lot expiration date. There were 14 patients screened and 4 patients did not meet the original inclusion criteria because they were not HLA-A2 positive. A protocol amendment allowed the enrollment of 3 HLA-A2 negative patients since tumor antigen-specific T cell assays were not the primary endpoint, and the HLA-A2 requirement was felt to delay subject accrual. One of the enrolled patients never received the study drug due to rapidly worsening pancreatitis. All 9 patients who received study drug are included in this analysis. Detailed patient characteristics are included in Table 1. The treatment group included 7 men and 2 women (8 Caucasian and 1 Hispanic) with ages ranging between 30–76 years. Most patients were stage IV M1c (6 of 9 patients), and 5 had baseline lactate dehydrogenase (LDH) levels above the upper limit of normal. All patients had received prior therapy for metastatic disease, which included IL2 (4 patients) and/or IFN-α2b (7 patients) in all patients based on the study eligibility of requiring prior cytokine-based therapy to participate in this study. Five patients had received prior chemotherapy for metastatic disease.

**Table 1:** Baseline characteristics of treated patients.

ID Number	Gender	Age (years)	KPS (%)	HLA-A2	LDH	Stage IV	Sites of Metastasis	Prior Cytokine Therapy	Prior Chemotherapy
0001–1103	M	49	80	+	169	M1b	Abdominal wall, thorax	IL2	Yes

0002–2101	M	30	90	+	130	M1b	Lung	IL2	No

0002–2102	F	39	80	+	429	M1c	Skin, lymph nodes, liver	IFNα2b	Yes

0003–3101	M	44	90	-	575	M1c	Skin, lymph nodes, lung	IL2 and IFNα2b	Yes

0003–3102	M	67	80	+	1388	M1c	Lymph nodes, skin, lung, liver	IL2 and IFNα2b	Yes

0004–4101	M	40	100	+	132	M1a	Skin	IFNα2b	No

0004–4102	M	36	90	+	333	M1c	Lymph nodes, spleen, liver	IFNα2b	Yes

0004–4103	F	54	90	-	94	M1c	Skin, liver	IFNα2b	No

0004–4104	M	76	90	-	282	M1c	Skin, lung	IFNα2b	No

ID = identification. KPS = Karnofsky performance status. LDH = lactate dehydrogenase (on Day 1 of Cycle 1). M = male. F = female. IFN = interferon. IL2 = interleukin-2.

### Study drug administration

Nine patients received the study drug. One subject (0002–2101) received a single cycle and withdrew from the study. Six patients received two cycles and 2 patients received 4 cycles of treatment. Subject 0002–2102 had a dose reduction due to the detection of an increase in liver enzymes after a single infusion in cycle 1. No further infusions were given for that cycle, and the patient received the 3 infusions of cycle 2 at a half dose (2 mg/m^2^/d). One subject (0004–4104) was overweight and was dosed at the ideal body weight rather than the actual body weight. The total cumulative dose administered ranged from 17.0 mg to 115.2 mg.

### Pharmacokinetics

**Figure 1:**
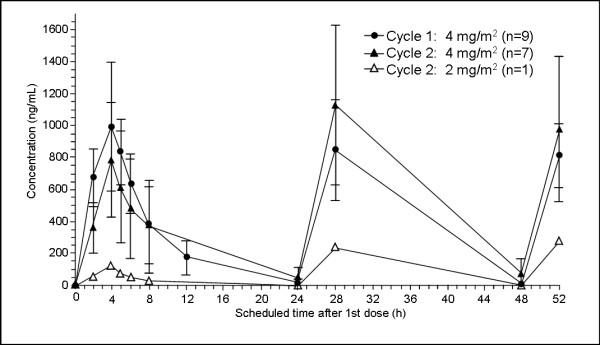
**Mean serum concentration-time profiles of EMD 273063**. Mean serum concentration-time profiles after daily 4-h infusions of EMD 273063, days 1–3 by cycle and treatment. Depicted are the mean serum concentrations (linear scale with SD) for patients in cycle 1 at dose 4 mg/m^2 ^(n = 9, closed circles), cycle 2 at dose 4 mg/m^2 ^(n = 7, closed triangles), and the one (n = 1, open triangles) patient who received cycle 2 at dose 2 mg/m^2^. Blood samples for PK analysis were drawn during cycles 1 and 2 as pre-dose and post-dose samples (2, 4, 5, 6, 8, 12, and 24 h, with the 12-h sample not taken in cycle 2). The 4-h time point corresponded to the end of infusion (EOI). Additional pre-dose and EOI samples were taken on days 2 and 3 of both cycles. An ELISA was used to measure EMD 273063 levels.

Serum concentration-time profiles of EMD 273063 were available from 9 patients during cycle 1 and 8 patients during cycle 2 (7 patients on 4 mg/m^2^/d and 1 patient on a reduced dose of 2 mg/m^2^/d). C_max _was achieved at the end of the 4-h infusion (Figure 1). Peak levels on days 2 and 3 of cycle 1 revealed no drug accumulation. Peak levels and extent of exposure (C_max _and AUC) decreased by approximately 30% on day 1 of cycle 2 compared with cycle 1, while the mean systemic clearance increased slightly from 1.26 L/h to 1.53 L/h. Data from both cycles indicated that the drug is cleared with an average half-life of 3.3 h (range: 1.6–8.2 h). In contrast to cycle 1, higher mean peak concentrations were observed on day 2 and 3 during cycle 2. This trend in accumulation was mainly based on the data of 3 out of 7 subjects (4101, 4102 and 4104) who showed quantifiable trough values that were in accordance with the prolonged half-life (4.7–8.2 h). Generally, variability in peak concentrations and derived pharmacokinetic parameters was higher during cycle 2 compared with cycle 1.

### Toxicity

As shown in Table 2, 6 patients experienced grade 3 or 4 adverse events. There were 2 patients with grade 4 adverse events: subject 0002–2102 experienced an increase in lipase without clinical evidence of pancreatitis, and subject 0003–3102 experienced urinary tract obstruction. Neither of these events was considered to be study drug-related. The most common grade 3 events were rigors (patients 0004–4101, 0004–4102, 0004–4104) and pyrexia (patients 0004–4101, 0004–4102), which are known to be associated with IL2-based therapy [11] and were attributed to the study drug. In addition, all patients experienced grade 1 or 2 IL2-related adverse events including nausea, rigors or pyrexia. Other common adverse events included vomiting (7 patients), fatigue (6 patients), flushing (6 patients), and pruritic rash (4 patients). Three patients developed edema, including periorbital edema, ankle edema, lymphedema, and/or pitting edema. IL2-related cardiovascular adverse events such as changes in blood pressure and heart rate were occasionally observed during the infusions, with the most consistent finding being an increase in heart rate. Mild hypertransaminasemia, which did not surpass 3 times the upper limit of normal, was observed.

**Table 2:** Dose intensity, Grade 3/4 adverse events and objective tumor responses.

ID Number	Number of Completed Cycles	Total Cumulative Dose (mg)	Grade 3/4 Adverse Events	Objective Response
0001–1103	2	46.8	None	PD

0002–2101	1	23.7	None	PD

0002–2102	2	17.0	ALP NOS increased Amylase increased Lipase increased Liver function tests NOS increased	PD

0003–3101	2	54.0	None	PD

0003–3102	2	45.1	Ureteric obstruction	PD

0004–4101	4	115.2	Pyrexia Rigors Hyponatremia Hypoxia	SD × 4 mo.

0004–4102	2	42.7	Pyrexia Rigors	PD

0004–4103	2	43.6	ALT increased Hypokalemia Rash NOS	PD

0004–4104	4	85.4	Arthralgia Rigors	SD × 4 mo.

PD = progressive disease. SD = stable disease. ALP = alkaline phosphatase. NOS = not otherwise specified. ALT = alanine aminotransferase.

### Clinical outcome

There were no major objective tumor responses. One patient (0004–4104) had stable disease for 4 months and another patient (0004–4101) had early progressive disease between cycles 1 and 2, followed by disease stabilization between cycles 2 to 4. Both patients had disease progression after 4 months. 6 other patients had disease progression at the first evaluation at the end of cycle 2 and were discontinued from therapy at that time, and one patient withdrew after one cycle.

### Immune monitoring in peripheral blood samples

**Figure 2:**
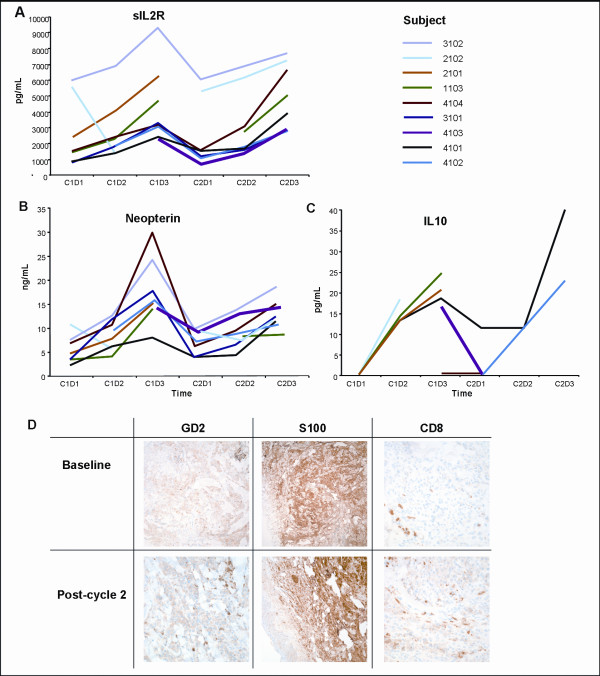
**Serum cytokine concentrations and immunohistochemical analysis of tumor biopsies**. C = cycle. D = day. A, B, C: Serum concentrations of sIL2R (A), neopterin (B) and IL10 (C) before, during, and after infusions of EMD 273063. Serum samples were drawn before the first infusion (C1D1), during the first cycle infusion (C1D2 and C1D3) and then immediately before (C2D1) and during the second cycle of EMD 273063 (C2D2, C2D3). Depicted are the serum concentrations for each patient tested by ELISA. D: Immunohistochemical analysis of a pre-dosing and cycle 1 post-dosing tumor biopsies from patient 4104, who had disease stabilization over two cycles. Paraffin-fixed melanoma tumor specimens stained by immunohistochemistry for GD2, S100, and CD8 positive prior to and after exposure to EMD 273063.

Exploration of biologic changes in post-dosing serum samples compared with baseline results demonstrated 3 parameters with statistically significant treatment-associated increases in the omnibus test: sIL2R (adjusted p < 0.0001), neopterin (adjusted p < 0.0003) and IL10 (adjusted p = 0.0345) (Figure 2A, B, C and Table 3). There were no changes in serum levels of S100 and IL6. There were also no significant changes in the frequency of CD4+ and CD8+ T cell subsets, NK cell number, NK activity, and ADCC between pre- and post-dosing blood cell samples. There was no difference between the 2 patients (0004–4101, 0004–4104) with stable disease who received 4 cycles of therapy and the 7 patients who progressed early with respect to changes in any of the parameters examined.

**Table 3:** Analysis of immunological parameters in peripheral blood.

Evaluation	Any Difference (Mack Skillings Test)		Paired Comparisons* (Signed Rank Test)	
	
	Raw P Value	Adjusted P Value	P Value of Cycle 1 Comparisons	P Value of Cycle 2 Comparisons
CD4+	0.0203	0.1827	-	-

CD8+	0.0207	0.1827	-	-

CD56+	-	-	0.1250	-

CD16+/CD56+	-	-	1.0	-

CD25+	0.3192	1.0	-	-

CD27+	0.0709	0.4254	-	-

NK+ granzyme B+	0.2623	1.0	-	-

CD8+ granzyme B+	0.0948	0.4740	-	-

NK activity	0.0207	0.1827	-	-

ADCC w/IL2	0.2818	1.0	-	-

ADCC	0.5095	1.0	-	-

S100	-	-	0.3621	-

IL6	0.0366	0.0732	-	-

sIL2R	<0.0001	<0.0001	0.0156	0.0156

TNF	0.1948	0.1948	-	-

IL10	0.0115	0.0345	0.0769	0.250

Neopterin	<0.0001	<0.0003	0.0156	0.0222

* For data with 3 or more serial observations, a Mack-Skillings test adjusted p < 0.05 was required to conduct paired comparisons. For selected parameters with too few observations, a paired (signed rank) test was conducted.

### Analysis of tumor biopsies

We compared tumor tissue specimens obtained at initial screening and approximately day 10 of the first 2 cycles. Table 4 shows that most biopsies were positive for GD2 and S100 prior to treatment with EMD 273063. Nearly all pre-treatment tissue specimens were negative for intratumoral lymphocytic infiltrates, but there was presence of CD16+ cells (a marker of macrophages and NK cells) in 6 of 7 specimens stained.

**Table 4:** Analysis of immunological parameters in tumor biopsies.

Evaluation	Pre-Dose Biopsy			Post-Dose Biopsy				
	
	Negative	Positive^1^	NA	Decreased^2^	No Change	Increased^2^	Increased, then Decreased	NA
GD2	2	5	2	3	3	0	1	2

S100	0	7	2	0	4	3	0	2

HLA-A	1	5	3	1	2	1	0	5

TIL by H&E	5	0	4	0	4	0	1	4

TIL by granzyme B	6	1	2	0	4	3	0	2

TIL by CD3	5	2	2	1	1	4	1	2

TIL by CD3zeta	5	2	2	1	3	3	0	2

TIL by CD8	5	2	2	1	3	3	0	2

TIL by CD16	1	6	2	0	6	1	0	2

TIL by CD56	6	0	3	0	4	0	0	5

Number of patients with negative and positive evaluations in pre-treatment (screen or day 1 of cycle 1) tumor biopsy specimens and changes in biology markers in tumor biopsies after exposure to EMD 273063 (1 or 2 cycles).

TIL = tumor infiltrating lymphocytes. H&E = hematoxylin and eosin. NA = not available.

1. ≥ 50% of cells with 1+ or greater staining intensity (GD2, S100, or HLA-A), or ≥ 1.0 cell/HPF (cells per high power field).

2. Increase or decrease determined by change in >10% cells (or an excess in changes of >10% of cells), or a change in number of cells/HPF from below to above or above to below 1.0, or from below to above or above to below 10.

After exposure to EMD 273063 there was a decrease in staining for GD2, the target of EMD 273063 on melanoma cells in 4 out of 7 cases studied (in one case the first biopsy showed an increase in GD2 staining, followed by a marked decrease in the second biopsy, and the other 3 cases showed no change; p = 0.125) (Table 4). There was a post-dosing increase in the staining intensity of tumor cells with the melanoma marker S100 in 3 out of 7 cases studied, and no change in the 4 other cases (p = 0.125) (Table 4). There was no obvious change for pan-HLA-A staining, which was included to evaluate the possibility of decrease of MHC expression as means of tumor escape. There was a trend towards increase in intratumoral CD3+ T cells and CD8+ T cells in most cases examined (Table 4). The IHC images from case 4104 are depicted in Figure 2D as a representative example of the post-dosing decrease in GD2 staining, and increase in S100 staining intensity and in CD8+ T cell infiltration. The EMD 273063-induced intratumoral lymphocytic infiltrates displayed increased staining for CD3zeta and granzyme B in 3 cases each (p = 0.3125 and p = 0.125, respectively by a one – tailed McNemars' test) (Table 4). However, this was a statistically non-significant finding, but was felt to be biologically significant since it follows the suspected mechanism of action of this immunocytokine. The small sample size and the known heterogeneity in immune responses make it difficult to assume that changes in intratumoral immune cell infiltrates would follow a statistically significant pattern. There were no differences in post-dose NK infiltration as detected by CD16 and CD56 staining (Table 4).

## Discussion

The purpose of this study was to explore the biologic and immunologic activity of the immunocytokine EMD 273063 and provide estimates for designing a future definitive study. We hypothesized that EMD 273063 would bind to GD2 on tumor cells; its IL2 moiety would then activate T and NK cells, which would release a secondary wave of cytokines, orchestrating an antitumor immune response. The main finding of this study was an increase in intratumoral CD8+ CTL with possible increased expression of CD3zeta and granzyme B after administration of EMD 273063. Since these results are based on a small sample size, they would require confirmation in a larger study.

Compared to the results from a previous phase I study with hu14.18-IL2 [7], peak concentrations and AUC values were only 1/3 of expected values. Since the half-life was in the same range in both studies, the clearance values obtained with the current study were higher. We do not have an obvious explanation for this finding, but several possibilities exist. In the phase I study, the peak serum levels of EMD273063 and AUC during course 1 showed a significant dose-dependent increase, whereas clearance showed a dose-dependent decrease. The dose used in the current study is 4 mg/m^2 ^which was between dose levels in the phase I study, so that our expected values could have been inaccurate. According to the phase I study, the presence or absence of macroscopic tumor does not influence the clearance of EMD 273063 [7]. In our study, the safety profile of EMD 273063 was consistent with the expected IL2 side effect profile as reported in the previous phase I clinical trial [7], except for a lower incidence of hyperglycemia and hypophosphatemia. Despite the intratumoral changes observed in our study of tumor biopsies, this clinical trial demonstrated no definitive antitumor activity with EMD 273063, which may be reflective of the small number and heavily pre-treated nature of the patients enrolled in this study or the small sample size with an inherently low probability (0.60) of observing even a single clinical response with 9 patients and an underlying response rate of 15%.

To gain insight on the effects of the immunocytokine on the immune system, we measured serum levels of immune-activating cytokines over the first 3 days of each treatment cycle. Our results show an increase in serum levels of sIL2R, IL10, and neopterin post-dosing. These findings may suggest the induction of both a T_h1 _response (sIL2Rα), monocyte activation (neopterin) as well as a T_h2 _response (IL10). Neopterin is produced in monocytes/macrophages upon stimulation with IFNγ and is commonly elevated in inflammatory conditions. Neopterin levels have been reported to be elevated following administration of IL2 [12], and our study demonstrates a similar increase with the administration of IL2 immunocytokines. The elevated IL10 could be evidence of monocyte stimulation or activation of Th2 cells since it is produced primarily by these cells. In contrast, we did not observe an increase in the percentage of CD16+ and CD56+ PBMC, an increase in NK lysis, or an increase in ADCC. These results should be interpreted with caution given that EMD 273063 has been previously shown to induce ADCC and NK cell-mediated lysis [7]. This discrepancy may be due to different techniques or the smaller sample size analyzed in our study. Regarding regulatory T cells (Treg), there was no comparable difference in the frequency of CD4 with CD25 staining (the phenotype of both T_reg _and activated T helper cells) comparing pre- and post-dosing samples. We did not have additional specimens for functional T_reg _determination, but this would be important to assess in further studies since IL2 has been shown to expand T_reg _[13] which could have a negative impact on the effector immune response activated by this immunocytokine.

The staining characteristics of the tumor cells suggest that the EMD 273063 immunocytokine had gained access to the tumor milieu. Although the choice of biopsy site and the random pathologic sampling in small specimens is likely to introduce variability not related to the treatment effect, exposure to EMD 273063 resulted in a decrease for 4 patients in GD2 staining on melanoma cells and increases in staining for S100. Whether this decrease in GD2 staining intensity represents antigen downregulation versus steric hindrance from the EMD 273063 bound to the tumor is not known. In studies of other anti-GD2 antibodies, conflicting results regarding internalization of the antibody (and presumably the GD2) have been observed with some showing that the GD2 remains on the surface [14] and others reporting internalization [15]. We also observed that some patients do not have GD2 expressed on their tumor and possibly these should be excluded in future studies. The explanation for increased S100 expression is also unclear; the 2 patients with stable disease did not demonstrate major changes in S100 intensity.

In this study, staining with a pan-HLA-A antibody did not change post-dosing, which suggests that tumor escape might not have been mediated through downregulation of MHC molecules after administration of EMD 273063. There was a trend towards an increase in intratumoral cell staining with the lymphocyte markers CD3 (total T lymphocytes) and CD8 (cytotoxic T lymphocytes), with possible increased staining for CD3zeta and granzyme B, effector molecules related to cytotoxic activity. However, there was no post-dosing change in NK infiltration as detected by CD16 and CD56 IHC staining. This observation is in contrast with findings in the peripheral blood that show no change in the number of lymphocytes that display CD3 and CD8 markers (total and CD8+ T cells, respectively), and may suggest that EMD 273063 effectively targets GD2 expressing tumors, and delivery of IL2 to the tumor microenvironment, resulting in expansion of CD8+ T cells, more notably CTL. It also supports the notion that the tumor may be a more appropriate site to study the interaction between the immune system and cancer cells, as opposed to the more common analysis of immune parameters in peripheral blood [16].

Whether directing IL2 to the tumor environment is the most appropriate way to enhance local immunity will require further study. Other approaches for introducing IL2 into the tumor environment include injection of the cytokine intratumorally [17] and administering intratumoral injections of adenovirus encoding IL2 [18]. In the later study, an objective response rate of 17% was observed for the injected lesions and stable disease was noted in some cases for non-injected lesions. In agreement with our study, they also noted increased intratumoral CD8+ T cells that were mainly of a cytotoxic phenotype, but minimal change in NK cell or CD4+ T cells. Similar results were observed for intratumoral injection of canarypox encoding IL2 [19]. These data suggest that intratumoral IL2 delivered by different strategies does result in enhanced CD8+ cytotoxic T cells intratumorally. Recently, intratumoral and intravenous immunocytokine administration was compared in murine models and the IT route [20] was more effective. Thus, future studies should evaluate the IT route in human tumors.

## Conclusion

In conclusion, EMD 273063 administered intravenously at 4 mg/m^2 ^daily for 3 consecutive days appears to be generally well tolerated with manageable toxicities, mainly expected IL2-related adverse events. Treatment with this agent is associated with immunologic effects as reflected by an increase in immune-related cytokines in serum and intratumoral changes in some patients consistent with increased intratumoral infiltration by CD8+ T cells. However, there was no apparent activation of NK function noted. Further studies looking at novel strategies aimed at enhancing immune activation by this immunocytokine to maximize antitumor responses are warranted.

## Competing interests

WG and TLW declare that they have no competing interests. AR is a speaker, consultant and/or receives grant support from: Amgen, Mannkind Corporation and Pfizer. JMK has received commercial research grants from Schering, BMS and Pfizer and served as a speaker for the Schering Plough Corporation. MBA has received commercial research grants from Novartis and Bayer/Onyx, served on Advisory Boards for Novartis, Antigenics, Schering and Medarex. AK and OK are employees of Merck KGaA; OK is also an Adjunct Professor at the University of North Carolina at Chapel Hill, NC, USA. SDG is a former employee of Merck KGaA and an inventor of patents related to hu14.18-IL2. MAM is a speaker, consultant and/or receives grant support form: Amgen, BMS, Bayer, Genentech, GlobeImmune, Immunitope, Novartis, Onyx, Roche, Sanofi-Aventis, Pfizer.

## Authors' contributions

AR participated in the study design and coordination and helped to draft the manuscript. JMK participated in the study design and coordination. MBA participated in the study design and coordination. TLW carried out and interpreted the immunoassay and drafted portions of the manuscript describing the assays. WG participated in the design of the study and performed the statistical analysis. AK participated in the study design and coordination. SDG participated in the study design and coordination. OK participated in the study design and coordination and helped to draft the manuscript. MAM participated in the study design and coordination and helped to draft the manuscript. All authors read and approved the final manuscript.
